# Postirradiation lumbosacral radiculopathy following seminoma treatment presenting as flaccid neuropathic bladder: a case report

**DOI:** 10.1186/1752-1947-5-148

**Published:** 2011-04-14

**Authors:** Omer A Raheem, David P Hickey

**Affiliations:** 1 Department of Urology and Transplantation, Beaumont Hospital, Dublin, Ireland

## Abstract

**Introduction:**

Postirradiation lumbosacral syndrome is a radiculopathy induced by radiation injury to the spinal cord. Its usual presentation is motor deficit and or sensory loss involving the lower limbs. Visceral involvement has not been reported previously.

**Case presentation:**

We describe a case of severe hypotonic bladder caused by radiation-induced spinal cord injury following treatment of stage Ι testicular seminoma in a 38-year-old Caucasian man who had undergone radical orchidectomy and prophylactic paraaortic lymph node irradiation for stage Ι seminoma. Three years later he had clinical and urodynamic findings of hypotonic bladder. The magnetic resonance imaging results suggested a radiation-induced injury.

**Conclusion:**

Such an unusual presentation of the syndrome of postirradiation lumbosacral radiculopathy can impose a clinical challenge to practicing clinicians. Future studies are required to further delineate the mechanism of injury and further management plans.

## Introduction

Postirradiation lumbosacral syndrome is a radiculopathy secondary to radiation injury of the spinal cord manifesting as motor and/or sensory loss involving the lower limbs. This case report describes a 38-year-old Caucasian patient with severe hypotonic bladder caused by radiation-induced spinal cord injury following treatment of stage Ι testicular seminoma. Our 38-year-old Caucasian patient had undergone radical orchidectomy and prophylactic paraaortic (PA) lymph node irradiation for stage Ι seminoma. Three years following radical orchidectomy and prophylactic PA lymph node irradiation, the patient presented with clinical and urodynamic findings of hypotonic bladder. The magnetic resonance imaging (MRI) findings suggested a radiation-induced injury. There is a paucity of systematic studies detailing the mechanism of injury of postirradiation radiculopathy, in particular bladder detrusor muscle involvement.

## Case presentation

A 38-year-old Caucasian man presented four years ago to our urology service with right-sided groin pain, and, on clinical examination, bilateral undescended testes were discovered. Clinically, the right testicle raised suspicions of malignancy, and a scrotal ultrasound confirmed a solid testicular mass. Tumor markers, including serum β-human chorionic gonadotropins, α-fetoprotein and lactate dehydrogenase, were normal. A right-sided radical orchidectomy was performed, and the left testicle appeared clinically healthy and was brought down into the left hemiscrotum and underwent successful three-point orchiopexy. Both the chest X-ray and abdominal computed tomography (CT) scan were negative for metastasis. The tumor histology results revealed an 8 cm seminoma confined to the testicle and stained positive for placental alkaline phosphatase. There were no syncytiotrophoblastic elements and no vascular or lymphatic invasion. The tumor was diagnosed as stage Ι testicular seminoma.

The patient received precautional PA field radiotherapy. Treatment was given five days per week for four weeks with daily fractions of 2 Gy up to a target dose of 25 Gy. The patient's follow-up protocol consisted of clinical evaluation, chest X-ray, tumor markers and an abdominal CT scan. The results of these investigations have remained normal to date.

**Figure 1:**
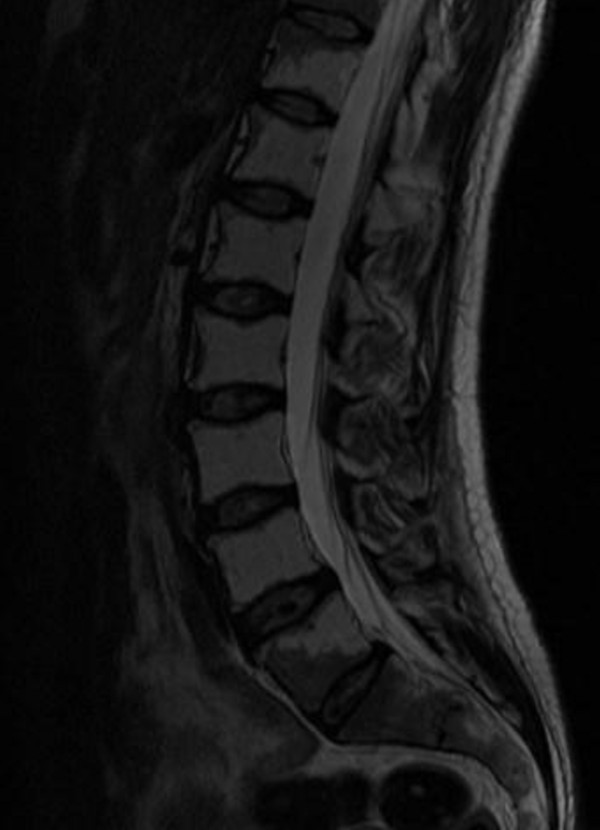
**T2-weighted, gadolinium-enhanced dorsolumbosacral magnetic resonance imaging scan showing an increased signal in the marrow fat at T11-L5 consistent with previous irradiation**.

Three years following prophylactic radiotherapy the patient presented with an episode of acute urinary retention mandating urinary catheterization. A full neurological examination did not reveal any abnormality. Urodynamic findings were a bladder capacity of 480 mL, absent detrusor instability and intact bladder sensation, but maximum detrusor pressure rose only to 11 cm/H_2_O at maximum capacity with an inability to micturate for voiding cystometry. A trial of pharmacotherapy with bethanechol chloride for four weeks at a dose of 10 mg thrice daily failed to improve both clinical and urodynamic findings. Consequently, the patient was commenced on clean intermittent self-catheterization, which he is managing well to date. A gadolinium-enhanced brain and dorsolumbosacral MRI scan showed in the lower two thoracic vertebrae (T11-T12) and all lumbar vertebrae (L1-L5) an increased signal in the marrow fat consistent with previous irradiation and with no obvious mass lesion in the spinal cord or vertebrae (Figure 1).

## Discussion

Testicular germ cell tumors (GCT) are the most common malignant tumors (17%) affecting men under the age of 45 years, with seminoma comprising 40% of GCTs. Prophylactic radiotherapy to the PA lymph nodes following orchidectomy for stage Ι seminoma provides a treatment modality that is the most cost-effective and is associated with the lowest risk of tumor recurrence (1% to 3%) [1].

Common side effects of radiotherapy include gastrointestinal toxicity, decreased sperm count and leukemia [1]. This case report describes an uncommon side effect of radiotherapy-induced injury following seminoma treatment [2].

The urinary bladder derives its innervations via the lumbar (sympathetic) and sacral (parasympathetic) regions of the spinal cord. A spinal cord lesion involving the lumbosacral roots typically presents as a lower motor neuron lesion with clinical and urodynamic findings of a flaccid neuropathic bladder [3].

Radiation-induced spinal cord injury principally involves the white matter. Several factors, such as radiation dose, fractionation or linear energy transfer, modify its occurrence and severity. The basic process for white matter injury as a result of irradiation involves radiation-induced vascular damage resulting in vascular hyperpermeability and venous exudation [4].

Postirradiation spinal cord injury is associated with long latency periods of up to 25 years following radiation treatment. The natural history of this disorder is one of relentless deterioration, occasionally punctuated by one to two year periods of stability [2]. Postirradiation radiculopathy has been reported previously. Neurological deficits include motor, sensory and occasional mild sphincter involvement [2,5,6].

In our patient, only the involvement of the urinary bladder and the absence of spinal cord or vertebral column masses on MRI scans clearly argue against metastatic disease. The urodynamic findings were those of a flaccid bladder, and the MRI findings suggest that the origin of the patient's spinal injury was radiation-induced. Our patient received a radiation dose of 25 Gy in accordance with the departmental treatment protocol. Although this radiation dose is generally considered to be a high dose according to current standards, it was effective and was associated with reduced side effects [7]. Currently, the clinical course of our patient is stable, with no further deterioration in the function of his urinary tract as proven by recent urodynamic study.

## Conclusion

A hypotonic bladder presenting suddenly in a patient who has undergone prophylactic radiotherapy for testicular seminoma may be the initial presentation of postirradiation lumbosacral syndrome.

## Consent

Written informed consent was obtained from the patient for publication of this case report and any accompanying images. A copy of the written consent is available for review by the Editor-in-Chief of this journal.

## Competing interests

The authors declare that they have no competing interests.

## Authors' contributions

Both authors read and approved the final manuscript.
